# Aortoiliac Occlusion Disease

**DOI:** 10.1055/s-0041-1729918

**Published:** 2022-06-02

**Authors:** Umberto G. Rossi, Anna M. Ierardi, Maurizio Cariati

**Affiliations:** 1 Department of Diagnostic Imaging, Interventional Radiology Unit, Ente Ospedaliero Galliera Hospital, Mura delle Cappuccine, Genova, Italy; 2 Advanced Technology Department of Diagnostic and Therapy, Radiology and Interventional Radiology Unit, Azienda Socio Sanitaria Territoriale Santi Paolo and Carlo – San Carlo Borromeo Hospital, Milano, Italy; 3 Department of Diagnostic Imaging, Radiology Unit, Istituto di Ricerca a Carattere Clinico e Scientifico Cà Granda Fondation, Maggiore Policlinico Hospital, Via Francesco Sforza, Milano, Italy

**Keywords:** aorta, iliac, artery, occlusion, vascular disease, imaging

## Abstract

Leriche syndrome is characterized by abdominal aorta and/or bilateral iliac occlusive disease, with a triad of clinical symptoms and signs such as claudication, erectile dysfunction, and decreased distal pulses. Diagnostic imaging is one of the key factors for diagnosis of the anatomic origin of the Leriche symptoms. We report the case of a 56-year-old man with diagnosis of abdominal aorta and bilateral iliac occlusive disease with a wide collateral vascular network.

A 56-year-old man, with a history of chronic hypertension and smoking, presented to our hospital for increasing symptoms for lower extremity intermittent claudication and impotence. Clinical examination revealed decreased femoral pulses and nonpalpable popliteal, dorsalis pedis, and posterior tibial pulses bilaterally. No vascular skin lesions were noted on the legs. The ankle–brachial indexes were markedly reduced, 0.45 on the right and 0.48 on the left. Leriche syndrome was diagnosed.

Multidetector computed tomography (MD-CT) with coronal volume rendering reconstruction ( Fig. 1 ) demonstrated abdominal aortic occlusion just below the origin of renal arteries (white arrowhead) with extension of nonvisualization to the bilateral common iliac arteries, confirming the origin of the Leriche diagnosis.

**Fig. 1:**
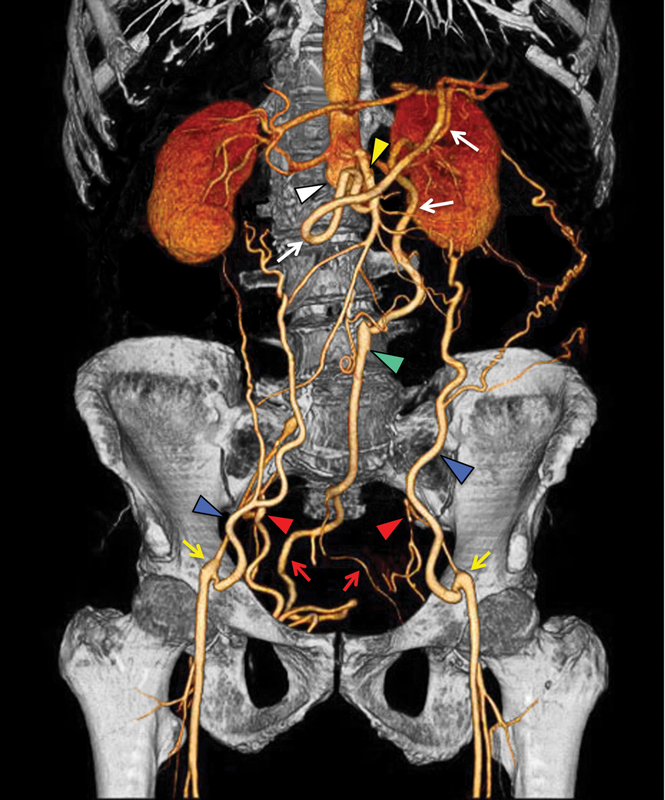
Multidetector computed tomography with coronal volume rendering reconstruction shows abdominal aortic occlusion below renal arteries origin's (white arrowhead) with extension to bilateral common iliac arteries. Note the hypertrophic network of collateral vessels: (1) superior mesenteric artery (yellow arrowhead) communicates with inferior mesenteric artery via Riolan's arc (white arrows), (2) inferior mesenteric artery (green arrowhead) through the superior rectal artery (red arrows) provides blood flow to internal iliac artery (red arrowheads), and (3) inferior epigastric arteries (blue arrowheads) guarantee blood flow to the bilateral external iliac arteries (yellow arrows).

A hypertrophic network of collateral vessels with revascularization to the bilateral iliac axis was highlighted. The following arteries formed collateral pathways: (1) superior mesenteric artery (yellow arrowhead) communicates with inferior mesenteric artery via Riolan's arc (white arrows), (2) inferior mesenteric artery (green arrowhead) through the superior rectal artery (red arrows) provides blood flow to internal iliac artery (red arrowheads), and (3) inferior epigastric arteries (blue arrowheads) guarantee blood flow to the bilateral external iliac arteries (yellow arrows). Based on these findings, the patient was a candidate for vascular surgery for aortobifemoral bypass grafting.

Abdominal aorta and bilateral iliac occlusive disease characterize as Leriche syndrome. A triad of symptoms/signs are seen: claudication, erectile dysfunction, and decreased distal pulses. The physiopathology results from obstructive atheromatous plaque formation at the level of the abdominal aorta and iliac arteries. The diagnosis is based on symptoms, ankle–brachial index, and diagnostic imaging. Angio MD-CT with three-dimensional reconstruction is the first-line diagnostic noninvasive imaging technique to evaluate aortoiliac disease. 1 2 3 4 This clearly demonstrates the extension of aortoiliac occlusion, arterial collateral pathways, and (with postprocessing) permits planning the correct treatment. 2 Treatment is focused on revascularization with either percutaneous endoluminal techniques or aortobifemoral bypass graft surgery.

## References

[1] Rossi U G Cariati M Aortoenteric fistula J Cardiovasc Comput Tomogr 2015 9 05 461 462 25958007 10.1016/j.jcct.2015.03.009

[2] Suh B Song Y S Shin D W Incidentally detected atherosclerosis in the abdominal aorta or its major branches on computed tomography is highly associated with coronary heart disease in asymptomatic adults J Cardiovasc Comput Tomogr 2018 12 04 305 311 29573979 10.1016/j.jcct.2018.03.001

[3] Rossi U G Ierardi A M Carrafiello G Cariati M Aortic coarctation Aorta (Stamford) 2020 8 02 46 47 32736405 10.1055/s-0040-1701522 PMC7394564

[4] Setacci C Galzerano G Setacci F Endovascular approach to Leriche syndrome J Cardiovasc Surg (Torino) 2012 53 03 301 306 22695262

